# Innovating Personalized Nephrology Care: Exploring the Potential Utilization of ChatGPT

**DOI:** 10.3390/jpm13121681

**Published:** 2023-12-04

**Authors:** Jing Miao, Charat Thongprayoon, Supawadee Suppadungsuk, Oscar A. Garcia Valencia, Fawad Qureshi, Wisit Cheungpasitporn

**Affiliations:** 1 Division of Nephrology and Hypertension, Department of Medicine, Mayo Clinic, Rochester, MN 55905, USA; miao.jing@mayo.edu (J.M.); thongprayoon.charat@mayo.edu (C.T.); supawadee.sup@mahidol.ac.th (S.S.); garciavalencia.oscar@mayo.edu (O.A.G.V.); qureshi.fawad@mayo.edu (F.Q.); 2 Chakri Naruebodindra Medical Institute, Faculty of Medicine Ramathibodi Hospital, Mahidol University, Samut Prakan 10540, Thailand

**Keywords:** artificial intelligence, chatbot, ChatGPT, nephrology, kidney disease, application

## Abstract

The rapid advancement of artificial intelligence (AI) technologies, particularly machine learning, has brought substantial progress to the field of nephrology, enabling significant improvements in the management of kidney diseases. ChatGPT, a revolutionary language model developed by OpenAI, is a versatile AI model designed to engage in meaningful and informative conversations. Its applications in healthcare have been notable, with demonstrated proficiency in various medical knowledge assessments. However, ChatGPT’s performance varies across different medical subfields, posing challenges in nephrology-related queries. At present, comprehensive reviews regarding ChatGPT’s potential applications in nephrology remain lacking despite the surge of interest in its role in various domains. This article seeks to fill this gap by presenting an overview of the integration of ChatGPT in nephrology. It discusses the potential benefits of ChatGPT in nephrology, encompassing dataset management, diagnostics, treatment planning, and patient communication and education, as well as medical research and education. It also explores ethical and legal concerns regarding the utilization of AI in medical practice. The continuous development of AI models like ChatGPT holds promise for the healthcare realm but also underscores the necessity of thorough evaluation and validation before implementing AI in real-world medical scenarios. This review serves as a valuable resource for nephrologists and healthcare professionals interested in fully utilizing the potential of AI in innovating personalized nephrology care.

## 1. Introduction 

Advancements in artificial intelligence (AI) technologies have notably influenced the landscape of various fields, including finance, transportation, and healthcare [1], leading to remarkable improvements in efficiency and productivity. AI is characterized by its capability to handle diverse tasks that traditionally need human intelligence. As AI applications become more integrated across different fields, the technology has grown to include major subsets like machine learning and deep learning. In medical research, machine learning has become particularly valuable for its ability to analyze and draw meaningful conclusions from the extensive data generated in healthcare. Specifically, in nephrology, a medical specialty focused on kidney diseases, machine learning has proven effective in classifying different patient subgroups across various kidney conditions, such as acute kidney injury (AKI), chronic kidney disease (CKD), end-stage kidney disease (ESKD), and kidney transplants [2,3,4,5]. Additionally, since 2018, there has been a surge in the utilization of machine learning-powered medical devices in healthcare. A thorough study revealed that a significant majority (77%) of these FDA-approved devices are predominantly used in radiology, with cardiovascular applications coming in second at 10% [6]. The most commonly approved types of these devices are radiological image processing and computer-assisted triage and notification systems [6]. However, it is noteworthy that in nephrology, the use of FDA-approved AI-enhanced medical devices is still an unexplored area.

ChatGPT, a significant language model introduced by OpenAI on 30 November 2022, is noted for generating human-like responses in a conversational style to user input [7,8]. Currently, ChatGPT has two primary versions: the broadly available GPT-3.5 and the more sophisticated, subscription-based GPT-4 [9]. ChatGPT exhibits its potential in various healthcare areas, including medical practices, research, and education [10,11]. Particularly notable is its remarkable proficiency in medical knowledge, which has been demonstrated to approach or even exceed the passing threshold (approximately 60%) for the United States Medical Licensing Examination (USMLE) [12,13]. In another study, ChatGPT nearly passed a radiology board-style examination with an accuracy of 69% [14]. In a third study, ChatGPT achieved an accuracy of approximately 80% in answering the questions from the competency-based medical education curriculum of microbiology [15]. However, it is important to consider the outcomes of an observational study that utilized the General Practitioners Applied Knowledge Test (GPAKT). The study revealed that ChatGPT’s overall performance was 60%, falling below the average passing threshold of 70% [16]. 

**Figure 1:**
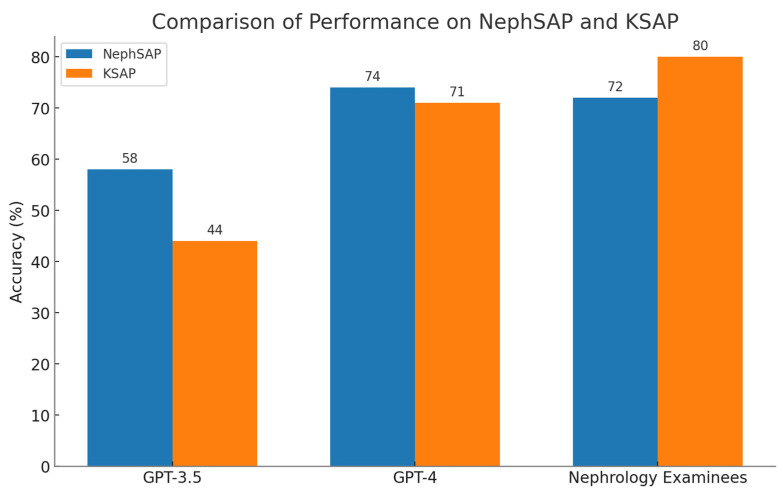
The performance of GPT-3.5, GPT-4, and nephrology examinees on NephSAP and KSAP test questions.

In our initial investigation, we observed that GPT-3.5’s performance was very limited when it came to responding to 150 questions related to glomerular disease from the Nephrology Self-Assessment Program (NephSAP) and Kidney Self-Assessment Program (KSAP). On the first and second runs, GPT-3.5 achieved an overall accuracy of only 44% and 41%, respectively. These results fell significantly below the required passing threshold of 75% set by the American Society of Nephrology (ASN) for nephrologists [17]. Subsequently, we conducted a comprehensive assessment using a larger set of 975 nephrology test questions, consisting of 508 questions from NephSAP and 467 from KSAP (Figure 1) [18]. GPT-3.5 obtained a total accuracy rate of 51% and a total concordance rate of 78%. Although GPT-4 showed improvement with a total accuracy rate of 74% and a total concordance rate of 83%, it still fell short of both the passing threshold and the average score of 77% achieved by nephrology examinees [18]. 

**Figure 2:**
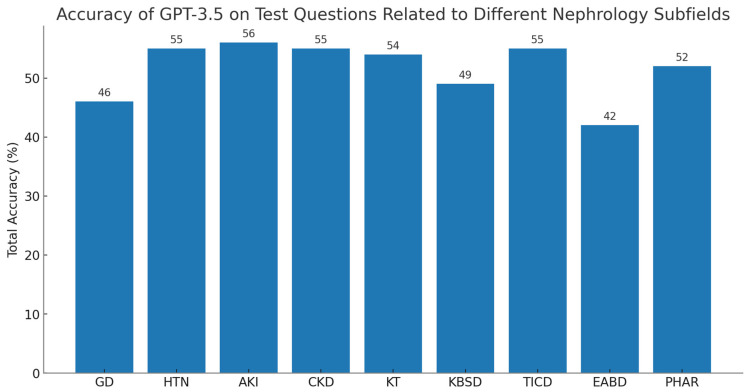
The performance of GPT-3.5 on test questions related to different nephrology subfields: GD: glomerular disease; HTN: gypertension; AKI: acute kidney injury; CKD: chronic kidney disease; KT: kidney transplant; KBSD: kidney-related bone and stone disorders; TICD: tubulointerstitial and cystic disorders; EABD: electrolytes and acid–base disorders; PHAR: pharmacology.

This comprehensive assessment also highlighted that ChatGPT’s performance varied notably across different subfields (Figure 2). As we observed, accuracy rates were relatively lower in areas such as electrolyte and acid–base disorders, glomerular diseases, and renal-related bone and stone disorders [18]. These subjects were more likely to require higher-level cognitive engagement, which ChatGPT is documented to be weaker at performing. For instance, clinical questions related to electrolyte and acid–base disorders require more complex calculations. This was also evident in glomerular diseases, where questions often require a detailed understanding of kidney pathology, physiology, and various treatment options, spanning a broad range of topics including immunology, genetics, and pharmacology. These results indicate that the AI model struggles more with tasks requiring in-depth understanding, analytical skills, and precise calculations. 

There has been a surge of interest in examining and discussing the potential practical uses of ChatGPT in various domains, which has mostly focused on its impact on medical education, scientific research, medical writing, ethical considerations, diagnostic or treatment decision making, automated data analysis, and criticisms [10,19,20,21,22,23,24], with over 1000 papers in PubMed dedicated to this subject. Yet, as far as our understanding goes, there is still a noticeable lack of a review consolidating the potential applications of ChatGPT, specifically within the field of nephrology, aiming to innovate personalized nephrology care.

## 2. Potential Applications of ChatGPT in Nephrology

**Figure 3:**
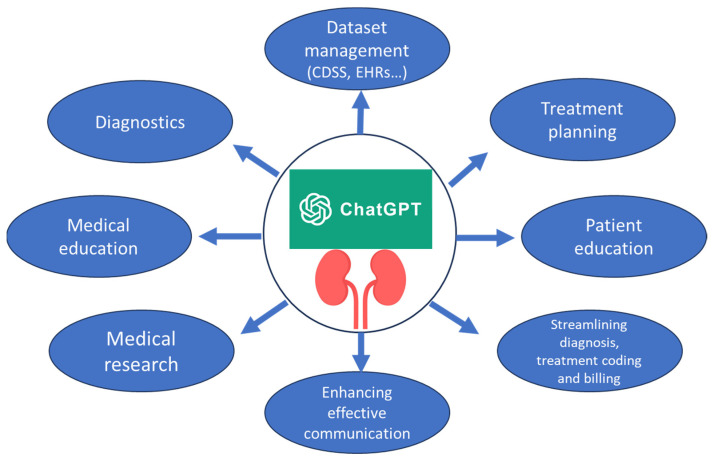
Potential applications of ChatGPT in the field of nephrology.

In nephrology, mastering the core principles and having access to appropriate datasets are vital elements for optimizing personalized patient outcomes and facilitating substantial research contributions. The following discussion will focus on the practical implications of integrating ChatGPT into nephrological clinical settings, as shown in (Figure 3). It is important to note that the GPT Builder represents a key feature of ChatGPT models [25]. One of the most significant advantages of the GPT Builder is its user-friendly interface, which does not require extensive programming knowledge. This accessibility means that nephrologists, researchers, and healthcare providers can directly involve themselves in the development and adaptation of AI models to suit their specific clinical and research requirements. The GPT Builder not only simplifies the creation and configuration of GPT models but also ensures that these models are more aligned with the specific needs of nephrology, thereby maximizing their effectiveness and utility in both clinical and research settings.

### 2.1. Integration of ChatGPT with Nephrology Datasets

#### 2.1.1. Current Datasets in Nephrology 

Electronic health records (EHRs) have revolutionized healthcare by providing a comprehensive digital repository of patient information [26]. In nephrology, EHRs capture clinical data related to kidney health, including laboratory results, imaging studies, medication history, and progress notes. EHRs offer several advantages, such as real-time availability of patient data, facilitating clinical decision making, and supporting research endeavors. By harnessing the wealth of information stored within EHRs, researchers and clinicians can conduct retrospective analyses, identify patterns, and generate evidence-based guidelines for optimal management of kidney diseases.

**Figure 4:**
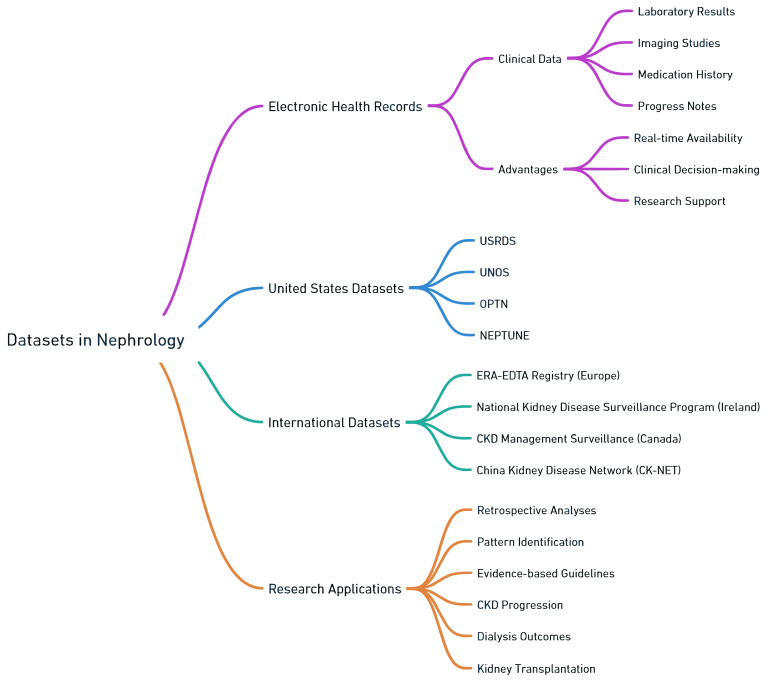
Overview of the datasets available for nephrology research.

Besides EHRs, several datasets specifically curated for nephrology research also provide valuable information on patient characteristics, disease profiles, treatment modalities, and outcomes [27]. Examples of widely used nephrology datasets (Figure 4) include the United States Renal Data System (USRDS), the United Network for Organ Sharing (UNOS), the Organ Procurement and Transplantation Network (OPTN), and the Nephrotic Syndrome Study Network (NEPTUNE). In addition to these databases in the United States, other countries worldwide also have big datasets within nephrology for researchers, such as the European Renal Association-European Dialysis and Transplant Association (ERA-EDTA) Registry, the National Kidney Disease Surveillance Program in Ireland, the surveillance project on CKD management in Canada, and the China Kidney Disease Network (CK-NET). These datasets offer valuable insights into diverse aspects of kidney disease, such as CKD progression, dialysis outcomes, and kidney transplantation.

#### 2.1.2. Implications for ChatGPT Integration 

While nephrology data sources offer immense potential, they are not without challenges and limitations. First and foremost is how to manage, handle, and process such a great amount of data that increased rapidly over time. Data quality and completeness can also be a significant concern. Inaccurate or missing data elements may limit the validity and generalizability of research findings. Furthermore, data interoperability and standardization issues across different healthcare systems can pose challenges in data integration and analysis.

Machine learning and other AI techniques possess the ability to handle intricate datasets and vast numbers of variables, surpassing the capabilities of classical statistical methods [28]. By leveraging AI tools like ChatGPT, the management of databases becomes significantly more feasible [29]. ChatGPT can be specifically fine-tuned to operate with a particular dataset and generate commands capable of executing various operations on that database. When incorporating ChatGPT or any other AI tool into nephrology data, it is essential to acknowledge and tackle potential biases and limitations associated with the model. Language models heavily rely on their training data, which can introduce inherent biases.

However, when applied appropriately, using ChatGPT for database management offers several advantages. Firstly, it saves valuable time and effort by automatically generating complex queries and commands, eliminating the need for manual and error-prone writing. Additionally, the fine-tuned model can be seamlessly integrated into larger applications, such as chatbots, enabling users to interact with the database using natural language. To ensure the accuracy and reliability of results, it is crucial to thoroughly evaluate and validate ChatGPT-generated outputs against expert knowledge and diverse patient populations. This evaluation helps mitigate potential biases. By addressing these challenges and leveraging available data effectively, researchers and healthcare professionals can lead to significant advancements in nephrology practice.

### 2.2. Applying ChatGPT in Nephrology Diagnostics

Accurate diagnosis is crucial for effective treatment and patient well-being. Human error can impede precise diagnostics due to the complexity and cognitive challenges of interpreting medical information. The advent of AI and natural language processing (NLP) technologies offers promising opportunities to revolutionize diagnostics [30].

#### 2.2.1. Integration of CDSS with ChatGPT in Nephrology

Clinical decision support systems (CDSSs) are computer-based tools designed to assist healthcare professionals in making clinical decisions and providing patient care [31,32]. These systems utilize patient-specific data, medical knowledge, and algorithms to provide recommendations, alerts, and reminders at the point of care. Although CDSSs offer numerous advantages, they also come with some drawbacks [31]. For instance, CDSSs can disrupt clinician workflows, especially in the case of stand-alone systems. Excessive and inappropriate alerts can burden clinicians with additional verification tasks and result in alert fatigue. Limited technological proficiency can hinder the effective utilization of CDSSs. A study indicated that ChatGPT-generated suggestions have the potential to serve as a valuable complement in the optimization of CDSS alerts [22,33]. They could play a significant role in assisting experts in developing their own suggestions for enhancing CDSS effectiveness. 

Additionally, many CDSSs lack transportability and interoperability, limiting their seamless integration into existing systems. While ChatGPT has limited ability to directly execute specific algorithms, it plays a valuable role in facilitating algorithm design for intelligent CDSS at the textual level [34]. One key aspect of integrating ChatGPT with CDSS is the seamless integration of the language model into the existing CDSS infrastructure. To optimize integration, ChatGPT should undergo fine-tuning specifically for nephrology-related tasks and queries. Real-time data integration from EHRs is also essential in the CDSS-ChatGPT system. By accessing up-to-date patient information such as laboratory results, medication history, and clinical notes, ChatGPT can generate more precise recommendations and assist clinicians in making informed decisions. Moreover, the integration can enhance clinical education and knowledge sharing by providing access to relevant literature, clinical guidelines, and case studies.

#### 2.2.2. Development of ChatGPT Diagnostic Model for Kidney Disease 

Building a ChatGPT diagnostic model specifically for kidney disease classification involves several crucial steps and considerations. Nephrology experts and data scientists need to collaborate to gather and curate diverse data from various sources like EHRs. These data include a wide range of kidney diseases, their associated symptoms, and relevant diagnostic criteria, as well as individual patient information (e.g., demographics, medical histories, laboratory results, imaging findings, and diagnostic outcomes). The dataset can be used to train ChatGPT to learn the relationships between input features and the corresponding kidney disease classifications, thus recognizing patterns and making accurate predictions. During the training process, the model’s performance is evaluated and adjusted by partitioning the dataset into training and validation subsets and further enhanced through techniques like transfer learning and fine-tuning. This ChatGPT diagnostic tool has the potential to assist healthcare providers in accurately identifying and classifying various kidney diseases, contributing to timely and effective treatment decisions [35]. 

#### 2.2.3. Assessing Performance of ChatGPT in Nephrology Diagnostics 

Evaluating the performance, including the accuracy and repeatability, of ChatGPT in nephrology diagnostics is crucial for assessing its reliability for clinical use. During an assessment involving a typical clinical scenario of acute organophosphate poisoning, ChatGPT-3 demonstrated commendable performance in addressing all inquiries, including making a diagnosis [36]. In a research study, the diagnostic accuracy of ChatGPT-3 was evaluated for ten common clinical cases with typical symptoms. The study showed that ChatGPT generated differential diagnosis lists with a high accuracy of 93%, although this accuracy rate was slightly lower compared to the physicians’ rate of 98% [37]. In another examination focused on evaluating the diagnostic capabilities of ChatGPT-3.5 in relation to kidney disease, the system exhibited an impressive accuracy rate of 91%, outperforming human physicians in both accuracy and speed [38]. ChatGPT, particularly GPT-4, showed the potential to provide faster responses to routine clinical laboratory questions. The study reported a correct rate of 76% (completely correct 51% and partially correct 23%), but the correct answers were most frequently seen in questions related to basic medical or technical knowledge [39]. 

Several important considerations should be taken into account during these evaluations to ensure robust and valid results. Firstly, a benchmark must be established for comparison. This can be achieved by using existing diagnostic methods or expert opinions as a reference standard. By comparing ChatGPT’s diagnostic performance against established standards, we can evaluate its accuracy, sensitivity, specificity, and overall diagnostic usefulness. To ensure generalizability and minimize bias, a comprehensive evaluation should involve a diverse range of patient cases covering various nephrological conditions and different patient demographics. Secondly, to quantitatively evaluate and compare the diagnostic accuracy of ChatGPT, it is recommended to employ performance metrics such as precision, recall, F1 score (combination of the precision and recall scores), and receiver operating characteristic (ROC) curves. These metrics offer objective measures of the model’s performance, enabling a comprehensive assessment of its strengths and limitations [40,41]. Prospective studies can be conducted to compare the diagnostic accuracy and treatment decisions made with and without the assistance of ChatGPT.

### 2.3. Applying ChatGPT in Treatment Planning

#### 2.3.1. ChatGPT-Powered Treatment Recommendations and Personalized Care 

**Figure 5:**
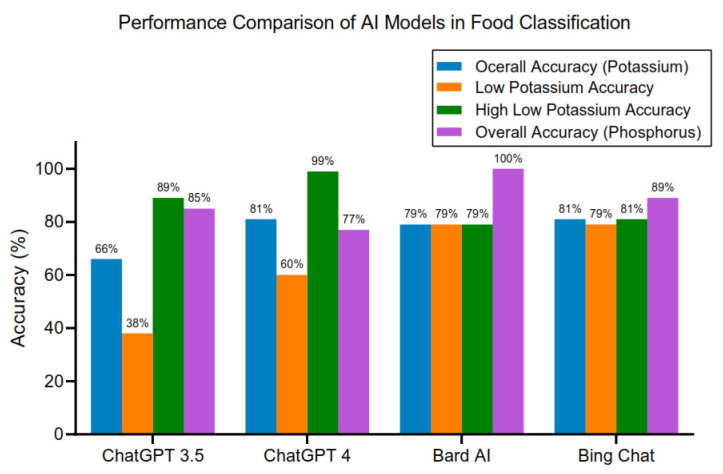
The performance of the four AI models in classifying food items based on their potassium and phosphorus content.

A significant benefit of incorporating ChatGPT into treatment strategies lies in its capacity for crafting individualized medical guidance [42,43,44]. Research shows that ChatGPT-4 has demonstrated proficiency in pinpointing medications adhering to established guidelines for treating advanced solid tumors [45]. By processing diverse patient data, including medical histories, lab test results, and coexisting health issues, ChatGPT can formulate personalized treatment options. These may involve specific drug dosages, recommended dietary changes, alterations in daily habits, and tailored monitoring protocols [46]. Right now, there exist two main types of leading chatbot models. One type revolves around OpenAI’s versions like ChatGPT-3.5, ChatGPT-4, and Bing Chat. Microsoft introduced Bing Chat in February 2023 as part of its search engine, utilizing OpenAI’s GPT-4 model for conversational AI features [47]. The other notable model is Bard AI from Google, which is based on the Pathways Language Model 2 (PaLM2), a transformer language model [48]. In a recent study evaluating the capability of ChatGPT-3.5, ChatGPT-4, Bard AI, and Bing Chat in discerning potassium and phosphorus content in foods for CKD patients, distinct levels of accuracy were observed (Figure 5). Using 240 food items from the Mayo Clinic Renal Diet Handbook, each model categorized foods as high or low in potassium and phosphorus, and their results were compared to the handbook’s guidelines. ChatGPT-4 led in potassium identification with 81% accuracy, excelling notably over ChatGPT-3.5, which had a 66% accuracy rate. In phosphorus identification, Bard AI achieved perfect accuracy, while ChatGPT-4 lagged with 77%. These findings highlight the burgeoning role of AI in facilitating renal dietary planning, though improvements are needed for maximum effectiveness.

#### 2.3.2. ChatGPT-Powered Decision Support for Medication Management

Medication management is a critical aspect of nephrology care, considering the complexity of drug regimens and the potential for medication-related complications. By integrating with EHRs and leveraging its vast knowledge base, ChatGPT can assist in drug–drug interaction checks, dosage adjustments based on renal function, and adherence monitoring. Furthermore, ChatGPT can facilitate communication between healthcare professionals and patients regarding medication-related queries, side effects, and treatment goals. The utilization of ChatGPT in a case report regarding a delayed diagnosis for a 27-year-old woman who reported chest pain and shortness of breath to the emergency department suggests its potential for clinical decision making, including improved diagnostic accuracy and addressing human factors contributing to medical errors [49]. Another study was conducted to see how well ChatGPT can answer medical questions [50]. The authors tested it with a case of a 22-year-old man who has a condition called treatment-resistant schizophrenia (TRS) and compared ChatGPT’s recommendations for assessment and treatment with the current standards used by doctors. The results showed that ChatGPT correctly identified the patient’s condition as TRS. It also suggested a thorough examination to find out the possible medical and psychiatric causes of the symptoms. Additionally, ChatGPT provided a comprehensive treatment plan, including medication and non-medication options, which matched the usual care provided by healthcare professionals for TRS. 

### 2.4. Integrating Patient Education and Counseling with ChatGPT 

Patient education and counseling are critical components of healthcare, aimed at empowering patients to actively participate in their treatment and manage their condition effectively. ChatGPT can serve as a valuable tool for integrating patient education and counseling into the nephrological treatment planning process [51], such as enhancing kidney transplant care [52].

Firstly, ChatGPT can provide accessible and personalized educational materials to patients. With its vast knowledge base, ChatGPT can offer comprehensive information on kidney diseases, treatment options, lifestyle modifications, and self-care practices [53]. Patients can engage in interactive conversations with ChatGPT, asking questions and seeking clarification on complex medical concepts. By delivering information in a conversational and user-friendly manner, ChatGPT can enhance patient comprehension and engagement with educational content. A study indicated that ChatGPT answered all queries well and offered good explanations of the underlying reasons regarding a typical clinical toxicology case of acute organophosphate poisoning retrieved from an online presentation [36]. The accuracy and reproducibility of ChatGPT were also examined in answering questions regarding knowledge, management, and emotional support for cirrhosis and hepatocellular carcinoma. While approximately 75% of the questions were answered with reliable information, the information regarding treatment recommendations such as decision-making cut-offs and treatment durations did not always align with the United States guidelines [54,55].

Secondly, ChatGPT can support counseling sessions by providing empathetic and supportive interactions. Patients living with kidney disease often face emotional and psychosocial challenges, including anxiety, depression, and uncertainty about the future. ChatGPT can act as a virtual companion, offering emotional support and guidance during counseling sessions. Patients can express their concerns, fears, and frustrations to ChatGPT, which can respond with empathy, validation, and practical advice. By facilitating open and non-judgmental conversations, ChatGPT can help patients navigate their emotional well-being and improve their overall quality of life. A cross-sectional study showed that ChatGPT generated quality and empathetic responses to patient questions posed in an online forum [56]. Moreover, ChatGPT can also help patients set realistic goals, develop personalized action plans, and track their progress. By providing reminders, motivational messages, and accountability, ChatGPT can support patients in making sustainable lifestyle changes and adhering to their prescribed treatment regimens [46]. The interactive nature of ChatGPT allows for ongoing communication, enabling patients to seek guidance and discuss challenges they encounter in their self-management journey.

### 2.5. Streamlining Diagnosis, Treatment Coding, and Billing with ChatGPT

ChatGPT presents the opportunity to enhance productivity and reduce expenses, thereby streamlining overall operations in healthcare services [57]. Proper coding and billing are essential for documenting diagnoses, procedures, and services provided to patients accurately. They enable healthcare providers to communicate essential information to payers, government agencies, and other stakeholders involved in reimbursement processes. Moreover, accurate coding and billing contribute to transparent and ethical financial practices in healthcare settings.

#### 2.5.1. Enhancing ICD-10 Diagnosis Coding with ChatGPT

ICD-10 coding is a complex process that requires adherence to specific guidelines and conventions for accurate diagnosis coding. The extensive range of kidney diseases, their variations, and associated comorbidities pose challenges in selecting the appropriate diagnosis codes. A study was conducted to develop an automated ICD-10 coding system using a deep neural network based on supervised learning. The researchers’ model demonstrated a notable improvement in the F1 score; however, it did not result in a reduction in the time required for disease coders to perform coding tasks [58]. ChatGPT’s NLP capabilities enable it to analyze patient information, laboratory results, imaging reports, and clinical notes in real-time, and provide immediate suggestions for diagnosis codes. Through continuous feedback and suggestions, ChatGPT can enhance coding accuracy, reduce the need for retrospective code correction, and minimize the potential for claim denials or payment delays. ChatGPT’s real-time coding assistance can also save time for healthcare providers, allowing them to focus more on patient care and reducing the administrative burden associated with coding tasks [59].

#### 2.5.2. Enhancing Treatment Coding with ChatGPT 

Current procedural terminology (CPT) codes provide a standardized system for identifying and reporting medical procedures and services. Nephrologists encounter various procedures in their practice, such as dialysis, kidney transplantation, vascular access placement, and kidney biopsies. Each procedure has a specific CPT code associated with it, reflecting the nature and complexity of the intervention. ChatGPT can provide coding support for these procedures by offering guidance on the appropriate CPT codes, documentation requirements, and coding modifiers that may be necessary [10]. For example, in the case of dialysis, ChatGPT can assist nephrologists in selecting the correct CPT codes for various dialysis modalities (e.g., hemodialysis, peritoneal dialysis, etc.) and associated services (e.g., vascular access management, dialysis catheter insertion, etc.). Similarly, in kidney transplantation, ChatGPT can aid in coding the transplant procedure, immunosuppressive therapy, and post-transplant follow-up care. Moreover, ChatGPT can offer real-time coding suggestions, ensuring that the appropriate CPT codes are assigned for the procedures performed. This can reduce the potential for coding errors and minimize the need for retrospective code corrections. 

#### 2.5.3. Improving Billing Efficiency with ChatGPT 

ChatGPT can analyze the clinical data within the EHR and assist in generating accurate billing codes, such as diagnosis-related group (DRG) codes, CPT codes, and evaluation and management (E/M) codes. This integration eliminates the need for manual code selection and reduces the potential for coding errors, leading to improved billing accuracy and efficiency [60]. In addition, this integration can offer the potential for enhanced communication between healthcare providers and insurance companies [60]. Through the generation of precise and standardized codes, the AI model enables smoother information exchange, leading to expedited claim processing and reimbursements. Furthermore, the integration of ChatGPT into billing processes can maximize revenue cycle efficiency by automating repetitive billing tasks, such as code generation, charge capture, and claims submission and identifying potential coding and billing discrepancies. This not only saves time but also reduces the risk of errors and improves overall billing accuracy [60]. Consequently, this can alleviate the administrative workload on healthcare providers, enabling them to dedicate more attention to patient care.

Utilizing ChatGPT and similar AI tools built on extensive large language models (LLMs) provides remarkable assistance in programming tasks, although their application necessitates careful consideration [61]. It is crucial to assess the influence of ChatGPT on coding accuracy, billing efficiency, and reimbursement to effectively determine its effectiveness in the field of nephrology practice.

### 2.6. Enhancing Effective Communication with ChatGPT

Effective communication is a cornerstone of quality healthcare delivery. Written communication plays a crucial role in conveying important information, facilitating collaboration, and advocating for patients. By utilizing ChatGPT, the field of medical writing has the potential to undergo a revolutionary transformation [62]. A systematic review indicated that the benefits of ChatGPT were most frequently cited in the context of academic and/or scientific writing, such as efficiency and versatility in writing with text of high quality, improved language, and good readability [10].

#### 2.6.1. Significance of Written Communication in Nephrology 

Written communication serves as a vital means of transmitting crucial information in nephrology practice [63]. It enables nephrologists to deliver comprehensive and well-organized messages, ensuring clarity and minimizing misinterpretation [64]. Letters are particularly valuable in conveying complex medical information to patients, referring providers, and payers, promoting understanding, collaboration, and continuity of care. Well-documented letters serve as a permanent record of patient assessments, treatment plans, and progress updates. These records not only support clinical decision making but also provide a legal and professional framework for healthcare providers to justify their actions and decisions.

#### 2.6.2. Utilizing ChatGPT for Crafting Patient Correspondence

When writing letters to patients, it is crucial to use language that is easily understandable and free of medical jargon. In the United States, it is recommended that patient-facing health literature be written at or below a sixth-grade level [65]. In addition, patient letters should provide educational resources (i.e., websites, brochures, or educational videos) to empower patients in self-management and address common questions and concerns that patients may have regarding their condition or treatment. ChatGPT can help craft patient-friendly letters to explain the diagnosis, treatment plans, and lifestyle recommendations using plain language and visual aids whenever possible. A pilot study showed that it is possible to generate clinic letters with a high overall correctness and humanness score with ChatGPT in a series of different clinical communication scenarios that covered the remit of a clinician’s skin cancer practice. Furthermore, these letters were written at a reading level that is broadly similar to current real-world human-generated letters [66,67]. 

#### 2.6.3. Leveraging ChatGPT for Communicating with Referring Providers

When communicating with referring providers, referral letters play a vital role in ensuring a seamless transition of care. Referral letters should provide a concise summary of the patient’s medical history, current condition, and the reason for referral and include relevant test results, imaging findings, and any specific questions or concerns that require attention.

In the context of referring letters, integrating ChatGPT can offer several benefits in updating referring providers on patient progress and recommendations. Firstly, ChatGPT can be utilized to automatically generate concise summaries of patient progress, extracting key information from EHRs and clinical notes. Secondly, ChatGPT can seamlessly integrate with CDSS to provide evidence-based treatment suggestions and recommendations. This integration ensures that referring providers receive accurate and up-to-date information regarding the patient’s progress. The suggestions can be included in the follow-up and consultation letters, ensuring that referring providers are informed about the most appropriate management strategies based on the latest medical knowledge. Thirdly, ChatGPT can assist in enhancing the language and clarity of the follow-up and consultation letters. By refining the language, ChatGPT can help nephrologists convey complex medical information in a more understandable and concise manner, facilitating better communication and comprehension for referring providers. In addition, ChatGPT can provide real-time assistance and feedback to nephrologists as they draft follow-up and consultation letters. By analyzing the content being written, ChatGPT can suggest additional details to include, prompt for clarification, or identify potential gaps in information. This interactive feedback helps nephrologists ensure that the letters are comprehensive and accurate before finalizing and sending them to referring providers. Furthermore, ChatGPT can support the implementation of shared decision making between nephrologists and referring providers. It can provide educational resources and relevant literature to facilitate discussions about treatment options, potential risks and benefits, and patient preferences. Ultimately, ChatGPT promotes a patient-centered approach and strengthens the therapeutic alliance between nephrologists and referring providers. 

#### 2.6.4. Utilizing ChatGPT for Medication Approval Appeals 

Medication approval appeals are essential when patients require specific medications that may not be initially approved by insurance providers or healthcare organizations. Writing medication approval appeal letters is a critical aspect of nephrology practice, as it involves advocating for patients’ therapeutic needs and ensuring that patients receive timely access to the medications required for managing their kidney disease and related conditions.

ChatGPT can provide valuable assistance in writing medication approval appeal letters. It can offer healthcare providers access to a vast array of medical literature, research articles, and clinical guidelines, enabling them to incorporate the most up-to-date and relevant information into their appeals. Additionally, ChatGPT can help refine the language, suggest alternative phrases, and ensure that the appeal letter adheres to the conventions of academic writing. By leveraging ChatGPT’s capabilities, healthcare providers can enhance the persuasiveness and effectiveness of their medication approval appeals, ultimately advocating for patients’ therapeutic needs.

### 2.7. Enhancing Nephrology Research with ChatGPT

#### 2.7.1. Leveraging ChatGPT for Data Analysis 

ChatGPT, as a powerful language model, can be used to gather data from large numbers of patients and extract valuable insights from unstructured textual data sources such as EHRs, clinical trial reports, and the scientific literature, streamlining the process of data collection and analysis [19,68]. This can be particularly useful in longitudinal studies, where ChatGPT can be used to track patient outcomes over time. One specific application of ChatGPT in nephrology research is the analysis of EHRs. By training ChatGPT on large-scale EHR datasets, researchers can develop models that can extract and analyze specific data elements relevant to nephrology, such as kidney function indicators, disease progression markers, and treatment outcomes. This enables researchers to gain deeper insights into the factors influencing kidney diseases, develop predictive models, and identify potential interventions for improved patient outcomes.

In addition, ChatGPT can generate commands and syntax for various statistical software packages, enabling researchers, even those with limited programming experience, to explore and analyze their data efficiently.

#### 2.7.2. Enhancing Articles Writing with ChatGPT

ChatGPT excels in accelerating the writing process, generating outlines, incorporating additional details, and enhancing the overall writing style [69,70,71,72,73,74,75]. The assistance of ChatGPT can be instrumental in identifying discussion points and clarifying language for the readers of medical literature reports [76,77]. However, it is important to acknowledge its limitations. As a precautionary measure, it is crucial to carefully review and edit the generated text to ensure that it adheres to ethical standards, avoiding issues like plagiarism and fabrication. 

#### 2.7.3. Assisting Nephrology Literature Analysis with ChatGPT

The vast amount of publications makes it challenging for researchers to keep up with the latest findings and integrate the existing knowledge effectively. Traditional literature review methods are time consuming and prone to human error. ChatGPT can aid in literature mining and analysis by automatically extracting relevant information from scientific articles, summarizing key findings and evidence, and identifying potential biases of relevant studies and relationships between different concepts. By leveraging ChatGPT’s capabilities, researchers can streamline the literature review process, identify knowledge gaps, and accelerate the discovery of new insights [78]. Its ability to handle large volumes of data and extract meaningful insights makes it a valuable tool for conducting rigorous systematic reviews and meta-analyses. 

**Figure 6:**
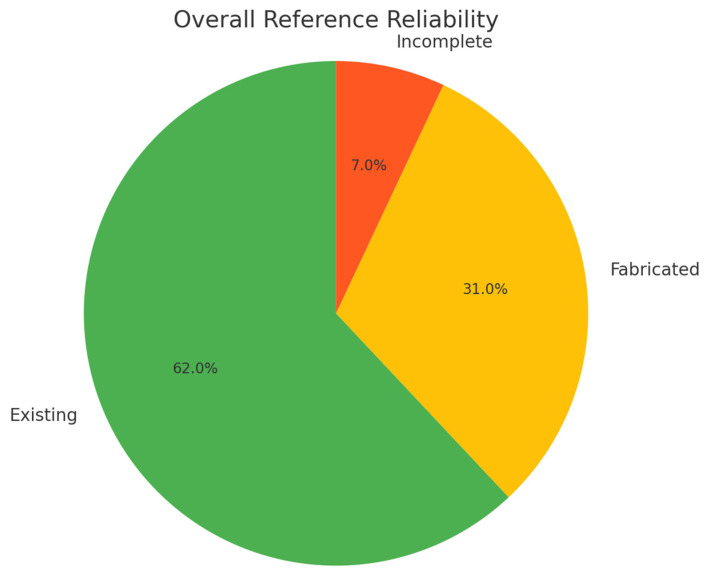
Overall Reference Reliability by ChatGPT 3.5.

We conducted an initial evaluation of ChatGPT’s performance in recognizing references related to literature reviews in the field of nephrology [79]. Our results showed that out of a total of 610 references, only 62% were accurately sourced by ChatGPT, while 31% were fabricated references, and 7% were incomplete citations (Figure 6). 

**Figure 7:**
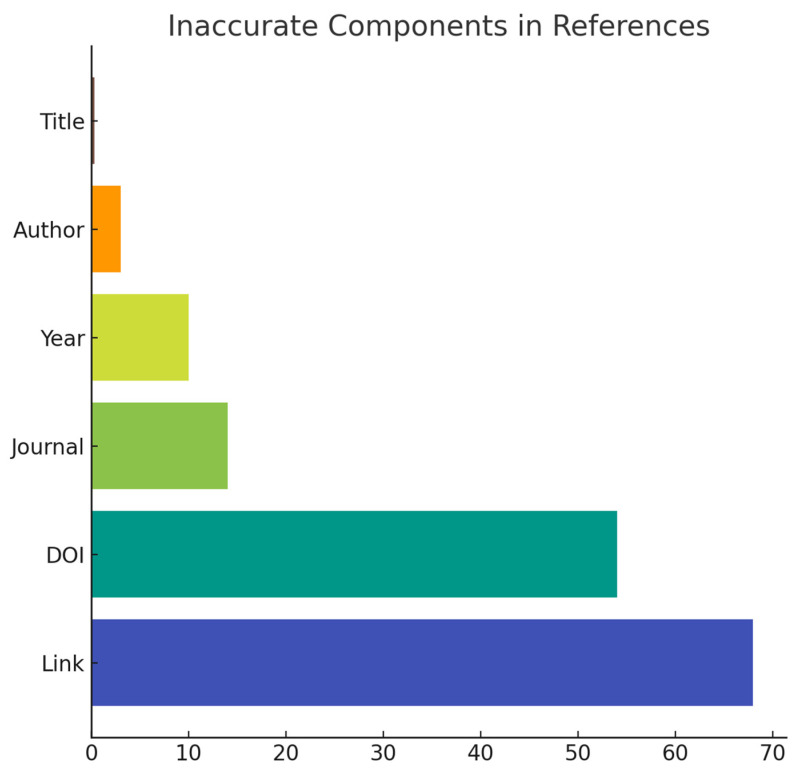
Inaccurate Components in References by ChatGPT 3.5.

Furthermore, approximately 70% of the provided links and half of the Digital Object Identifiers (DOIs) were found to be inaccurate (Figure 7). Notably, when we examined specific topics such as electrolyte balance, hemodialysis, and kidney stones, we found that over 60% of the references were either incorrect or misleading, often containing unreliable authorship and links. 

**Figure 8:**
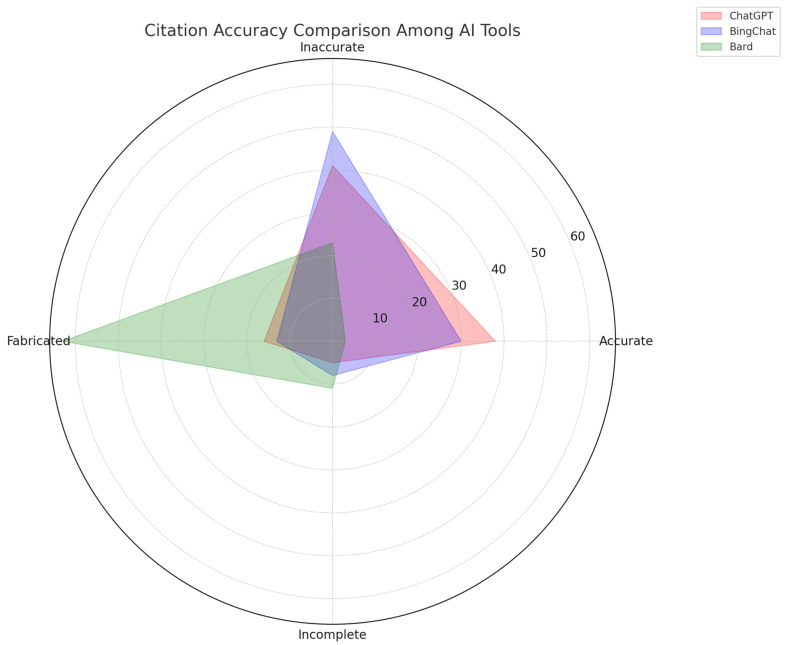
The radar plot provides a visual comparison of the citation accuracy of ChatGPT 3.5, BingChat, and Bard AI as of 1 August 2023.

Moreover, we recently evaluated the citation accuracy of AI tools, specifically ChatGPT, Bing Chat, and Bard AI, in the field of nephrology [80]. The evaluation involved generating prompts for each tool to provide 20 references in Vancouver style across 12 topics, followed by validation through PubMed, Google Scholar, and Web of Science. The findings reveal that Bard AI was the least accurate, providing only 3% accurate references along with a high percentage of fabricated (63%) and incomplete (11%) citations (Figure 8). Bing Chat performed marginally better but was still inadequate, with 30% accurate, 49% inaccurate, 13% fabricated, and 8% incomplete references. The most frequent error across platforms was incorrect DOIs. 

In light of our findings, it is advisable not to solely rely on ChatGPT for identifying references to literature reviews in the nephrology field at this time. Therefore, as with any automated tool, evaluating the reliability and accuracy of ChatGPT for literature review is of utmost importance. Researchers and developers need to assess its performance against established benchmarks and human reviewers. This involves conducting comparative studies to evaluate its ability to identify relevant articles, extract key information accurately, and provide reliable summaries. Additionally, assessing the robustness of ChatGPT in handling different study designs, languages, and data sources is crucial for determining its generalizability and applicability in the field of nephrology.

### 2.8. Enhancing Nephrology Education with ChatGPT

The successful completion of examinations like the USMLE by ChatGPT brings attention to certain shortcomings within medical education, particularly its heavy reliance on memorization rather than the analysis of intricate health and disease models. This accomplishment serves as a crucial reminder to re-evaluate the methods used to train and evaluate our medical students [81]. It is important to acknowledge that ChatGPT lacks the nuanced reasoning abilities possessed by humans. Consequently, it is imperative to recognize that AI can never replace the invaluable role played by nurses, doctors, and other healthcare professionals on the frontlines. However, there is no denying that AI and LLMs will revolutionize all aspects of our work, spanning from research and writing to medical diagnosis, treatment, and education across various fields [81,82,83,84]. 

#### 2.8.1. ChatGPT as an Educational Tool: Facilitating Learning and Knowledge Dissemination 

With its ability to generate human-like responses and engage in interactive conversations, ChatGPT can simulate real-time interactions between students and virtual tutors or instructors. This enables learners to ask questions, seek explanations, and receive instant feedback, thereby enhancing their understanding of complex nephrological concepts. Moreover, ChatGPT can adapt its responses based on the learner’s level of knowledge, providing personalized and tailored explanations to address individual learning needs.

Traditional educational materials, such as textbooks and lectures, may have limitations in terms of availability and accessibility. However, ChatGPT can be deployed as a web-based or mobile application, allowing learners to access educational content anytime and anywhere. Learners can engage in interactive discussions, explore case studies, and receive guidance on nephrology topics, ultimately fostering a self-directed and flexible learning experience. Furthermore, ChatGPT can also assist in knowledge dissemination within the nephrology community. It can be used to develop virtual educational platforms where nephrology experts can share their expertise, engage in discussions, and disseminate the latest research findings. By leveraging ChatGPT’s natural language generation capabilities, educational content such as question banks, tutorials, case studies, and guidelines can be generated and shared with a wider audience [85]. This not only promotes collaboration and knowledge exchange among healthcare professionals but also ensures that the most up-to-date information is readily available to support evidence-based practice.

#### 2.8.2. Integration of ChatGPT into Continuing Medical Education (CME) Programs

By incorporating ChatGPT into CME platforms, clinicians can access up-to-date research findings, summaries, and interactive discussions on relevant nephrology topics. This integration allows for personalized and on-demand learning experiences, enabling clinicians to stay informed about the latest advancements in the field. Moreover, ChatGPT can serve as a virtual assistant during CME activities, answering questions, providing clarification, and facilitating case-based discussions. The ability of ChatGPT to optimize and summarize the medical conference panel recommendations was assessed in the first Pan-Arab Pediatric Palliative Critical Care Hybrid Conference [86]. The results suggest that ChatGPT-4 effectively facilitated complex do-not-resuscitate (DNR) conflict resolution by summarizing key themes such as effective communication, collaboration, patient- and family-centered care, trust, and ethical considerations and demonstrated its potential benefits for enhancing critical thinking among medical professionals [86]. 

## 3. Ethical and Legal Implications of ChatGPT Integration in Nephrology

**Figure 9:**
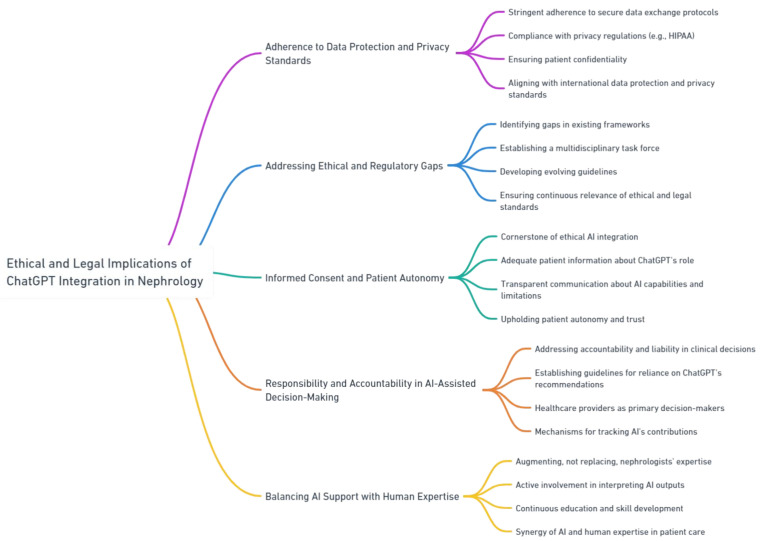
Ethical and legal implications of ChatGPT integration in nephrology.

Adherence to secure data exchange protocols and privacy regulations is crucial for ensuring patient confidentiality during clinical practices [87,88]. It is important to identify and address any potential ethical or regulatory concerns regarding the use of ChatGPT. At present, the norms and protocols governing the clinical implementation of AI are inadequately defined or non-existent [89]. Even so, the integration of ChatGPT in nephrology firstly requires compliance with existing regulatory frameworks and ethical guidelines, such as the Health Insurance Portability and Accountability Act (HIPAA) in the United States. Secondly, obtaining informed consent is imperative to ensuring that patients willingly consent to the utilization of AI-assisted healthcare (i.e., diagnostics or treatment planning) and have the autonomy to decline participation if they choose [90] (Figure 9). 

Additionally, integrating ChatGPT in nephrology raises questions about accountability and liability in decision-making processes [91,92]. While ChatGPT can provide valuable insights and recommendations, the ultimate responsibility for patient care lies with healthcare providers. Clear guidelines should be established to outline the roles and responsibilities of healthcare providers when using ChatGPT in clinical practice. It is crucial to define the extent to which healthcare providers rely on ChatGPT recommendations and how they integrate these recommendations with their own clinical expertise and judgment. Mechanisms for tracking and documenting ChatGPT’s contributions to clinical decision making should be established to ensure transparency and accountability. 

Moreover, the integration of ChatGPT should aim to enhance, rather than replace, the expertise of nephrologists. Healthcare providers must maintain an active role in interpreting ChatGPT outputs, critically evaluating recommendations, and making informed decisions based on the patient’s unique clinical context. Nephrologists should continuously update their knowledge and skills to effectively utilize ChatGPT as a supportive tool. The ethical integration of ChatGPT requires striking a balance between AI support and human expertise [24,93], ensuring that patients receive the highest quality of care that combines the benefits of AI technology and the human touch.

## 4. Future Directions and Challenges in Nephrology with ChatGPT

ChatGPT explores the latest developments in NLP, machine learning, and AI algorithms that have the potential to revolutionize nephrology practice in various ways [10,24,94]. In the future, collaboration between nephrologists and AI specialists is essential for optimizing the performance and impact of ChatGPT in nephrology practice, where nephrologists provide domain expertise and clinical insights while AI specialists contribute technical expertise in machine learning and NLP. By working together, these professionals can refine ChatGPT’s algorithms, develop specialized models tailored to nephrology-specific tasks, and integrate feedback mechanisms for continuous improvement. Collaboration also enables the customization of ChatGPT to address the unique challenges and complexities of nephrology, ultimately enhancing its clinical utility and effectiveness to provide personalized patient care. Additionally, we emphasize the importance of developing strong protocols for data collection, storage, and sharing to protect privacy and guarantee the security of data [95,96]. Furthermore, regulatory bodies, such as medical boards and professional associations, should start to establish the guidelines and ensure that ChatGPT applications meet the required standards for safety, accuracy, and reliability.

## 5. Conclusions 

ChatGPT holds immense potential to revolutionize nephrology practice by facilitating clinical decision making, enhancing patient communication, streamlining research, and improving operational efficiency. By embracing a multidisciplinary approach, fostering collaboration between nephrologists and AI specialists, and prioritizing ethical considerations, the future of ChatGPT in nephrology appears promising. Continued research, development, and evaluation will shape the evolution of ChatGPT, leading to its wider adoption and integration into routine nephrology care and ultimately improving patient outcomes and advancing personalized patient care in the field. However, it is essential to acknowledge that with the ongoing evolution of AI, it becomes particularly vital for the upcoming generation of physicians to adeptly navigate these challenges. They must weigh the potential benefits and risks to effectively determine how extensively AI should be integrated into medical practice.

## Data Availability

The data used in this study can be obtained upon reasonable request to the corresponding author.
